# Relevance of New Definitions to Incidence and Prognosis of Acute Kidney Injury in Hospitalized Patients with Cirrhosis: A Retrospective Population-Based Cohort Study

**DOI:** 10.1371/journal.pone.0160394

**Published:** 2016-08-09

**Authors:** Puneeta Tandon, Matthew T. James, Juan G. Abraldes, Constantine J. Karvellas, Feng Ye, Neesh Pannu

**Affiliations:** 1 Cirrhosis Care Clinic, Division of Gastroenterology, University of Alberta, Edmonton, Alberta, Canada; 2 CEGIIR, Department of Medicine, University of Alberta, Edmonton, Alberta, Canada; 3 Division of Nephrology, University of Calgary, Calgary, Alberta, Canada; 4 Division of Critical Care Medicine, University of Alberta, Edmonton, Alberta, Canada; 5 Division of Nephrology, University of Alberta, Edmonton, Alberta, Canada; Emory University, UNITED STATES

## Abstract

**Background:**

The implementation of new serum creatinine (SCr)-based criteria for acute kidney injury (AKI) has brought to light several areas of uncertainty in patients with cirrhosis.

**Study Design:**

Population-based cohort study.

**Setting & Participants:**

Adults with cirrhosis hospitalized between 2002–2012.

**Predictor:**

We aimed to address the prognostic implications of the new AKI criteria in cirrhosis.

**Outcomes:**

Baseline kidney function was defined from all outpatient SCr within 3 months before hospitalization. Cox proportional hazards models were fit to examine associations between AKI, renal recovery and all-cause mortality.

**Results:**

4,733 patients were studied. The 30-day mortality was higher for participants with AKI (43.9% vs 8.5%; p-value<0.001), and increased with AKI severity. The highest incidence of AKI occurred when the lowest SCr within the three months prior to admission was used to define baseline. The hazard ratio for mortality using the lowest SCr within 3 months and the closest pre-admission SCr (definition suggested by the recent consensus guideline) were similar, validating the use of the latter measure. As compared to patients without AKI, stage 1 AKI with maximum SCr ≤132 mmol/L remained associated with a 3.5-fold increased hazard of death at 30 days (95% CI 2.6 to 4.7).

**Limitations:**

As an observational study, the results were vulnerable to residual confounding and ascertainment bias in the use of laboratory data to identify AKI. We did not have access to liver function or disease etiology variables and were unable to adjust for these in our analyses.

**Conclusions:**

These results confirm the graded relationship between AKI severity, renal recovery, and mortality and further clarify previously discordant reports about the prognostic relevance of new AKI criteria in patients with cirrhosis.

## Introduction

Acute kidney injury (AKI) is diagnosed in 20–49% of hospitalized patients with cirrhosis [1–3] and is estimated to increase the 1-year odds of death by almost 8-fold as compared to cirrhotic patients without AKI [4]. In keeping with advances in nephrology, the definition of AKI in cirrhosis has shifted from requiring an absolute threshold serum creatinine (SCr) of ≥ 132.6 μmol/L (without consideration of the patient’s baseline SCr) [5] to recognizing the prognostic relevance of defining AKI based on relatively small increases in SCr compared to baseline. Several studies in cirrhotic populations have supported the transition to the novel criteria, reporting a graded association between AKI and adverse clinical outcomes [1–3, 6]. However, the implementation of these criteria has brought to light several areas of uncertainty [7].

First, defining AKI in the context of cirrhosis poses some challenge with respect to determination of baseline kidney function, as progressive hepatic decompensation is frequently associated with worsening kidney function. Existing studies, including the latest expert based consensus recommendations [7] have used varying definitions [2, 8, 9] with implications for the sensitivity to detect AKI [6, 10] and the estimation of prognosis. Secondly, although it is clear that AKI increases mortality, two recent studies have raised a debate as to whether the subtle differentiation into AKI stage 1 (an increase in the SCr of >26.5μmol/L over 48 hours) is relevant for the prediction of mortality in cirrhosis [8, 9, 11–13]. Lastly, although AKI has clearly been associated with increased mortality, little is known about the impact of renal recovery on this outcome in cirrhosis.

Using a population-based cohort of hospitalized adults with cirrhosis, we aimed to address these areas of uncertainty, adding to the observations from recent excellent studies [2, 8, 9, 14] and guidelines [7, 15] in the area.

## Materials and Methods

### Study population

The retrospective study cohort included all subjects residing in Alberta admitted to hospital with a diagnosis of cirrhosis from November 1, 2002 and March 31, 2012 with at least one outpatient SCr measurement within 90 days before hospitalization and ≥ 1 measurement during the hospitalization. If participants had >1 cirrhosis hospitalization episode during this period, only the first was considered. Participants with end-stage renal disease (ESRD-baseline estimated glomerular filtration rate (eGFR) <15ml/min/1.73m^2^, chronic dialysis, prior renal transplant) or liver transplant prior to hospitalization were excluded. All participants in the cohort had follow-up data until March 31, 2013. Data were drawn from hospital discharge abstracts of Alberta Health, the Northern and Southern Alberta Renal Programs, and the provincial laboratories of Alberta via the Interdisciplinary Chronic Disease Collaboration (ICDC) dataset [16]. Sequential SCr values are captured by this dataset and form the basis of the diagnosis of AKI and renal recovery defined below. The study was approved by the Health Research Ethics Board at the University of Alberta (project ID Pro00046970). As a retrospective study of de-identified administrative data, the Ethics Board did not require us to obtain written or verbal informed consent from participants.

### Identification of cirrhosis associated hospitalization

Cirrhosis associated hospitalization was defined using a validated algorithm [17]. All patients had ≥ 1 validated ICD-10 cirrhosis code. A sensitivity analysis was performed in patients with ≥ 2 ICD-10 codes [18].

### Assessment of baseline kidney function

SCr measurements within the province were standardized across provincial laboratories to an isotope dilution mass spectrometry reference standard, and a laboratory-specific correction factor applied when necessary [16]. The Chronic Kidney Disease Epidemiology Collaboration prediction equation was used to determine the eGFR [19]. Baseline kidney function was defined as the mean of all outpatient SCr in the 3 months before the index hospitalization. In sensitivity analysis, alternate definitions of baseline kidney function were considered: using the lowest SCr within 3 months, the mean of all outpatient SCr in the 6 months before hospitalization, the closest SCr to admission (in keeping with recent revised consensus recommendations) [7] and the first SCr during hospitalization.

### Identification of AKI

As per the KDIGO (Kidney Disease Improving Global Outcomes) criteria [20], AKI was defined as an increase in SCr from baseline by 26.5μmol/L within 48 hours of hospitalization or an increase in SCr to ≥ 1.5 times baseline during the index hospitalization. Severity of the AKI event was identified by the changes between baseline and peak in-hospital SCr and determined by the maximum KDIGO (Kidney Disease Improving Global Outcomes) [20] AKI stage reached during hospitalization.

### Assessment of comorbid conditions

Demographic characteristics including age, sex and comorbidity were determined from Alberta Health administrative data. Comorbid conditions were identified based on validated International Classification of Diseases, Ninth Revision (ICD-9) and Tenth Revision (ICD-10) coding algorithms [18] applied to hospital discharge abstracts and physician claims data. The burden of comorbidity was measured using the Charlson comorbidity score [18] and adjusted for in all multivariable analyses. Data required to calculate the MELD and Child Pugh score were not available in the dataset.

### Assessment of mortality

All-cause mortality was determined within 30 days after the index hospital admission date. A sensitivity analysis was performed to examine 90-day mortality (data in tabular format). All-cause mortality was identified using administrative data sources (provincial vital statistics).

### Assessment of renal recovery after AKI

Post-AKI renal function was assessed using the SCr drawn closest to 30 days after the onset of the AKI episode (recovery SCr). A recovery period of 30 days was chosen due to the high mortality observed in cirrhotic patients. Recovery was defined as a SCr within 25% of the baseline (prehospitalization value) and independence from renal replacement therapy. This definition of renal recovery was chosen as it is based on recommendations by the Acute Dialysis Quality Initiative [21], is consistent with contemporary definitions of AKI [21, 22] and has previously demonstrated clinical relevance [23].

The associations between renal recovery and mortality were assessed with follow-up for mortality to 6 months after the index hospital admission. A sensitivity analysis was performed with follow-up for mortality to 12 months after hospital admission, with results reported in tabular format.

### Statistical analysis

Continuous variables were described using the mean and standard deviation or median with interquartile range as appropriate. Categorical variables were described as proportions of the cohort, with between-group comparisons performed using chi-squared tests, ANOVA or Kruskal-Wallis as appropriate.

Multivariable Cox proportional hazard models with time varying covariates were used to estimate the associations between AKI, renal recovery and mortality. All clinically relevant variables, including age, gender, comorbid conditions, procedures or conditions during hospitalization, and baseline eGFR, were included in the models regardless of their significance in order to control for confounding. Time of origin was the date of admission for the index hospitalization. The proportional hazard assumption was evaluated and satisfied by examining plots of the log-negative-log within-group survivorship functions versus log-time.

In the models fit for assessing the association between AKI and mortality, AKI episodes (the time of peak in-hospital SCr) were used to update the exposure status during the course of follow-up, meaning that a person experiencing an AKI episode would contribute person time to the no AKI group hazard before their AKI episode and person time to the hazards of AKI stage 1, 2, or 3 groups after their AKI episode. Patients were censored if they moved out of the province, or reached 30 days/90 days from the time of origin.

**Fig 1:**
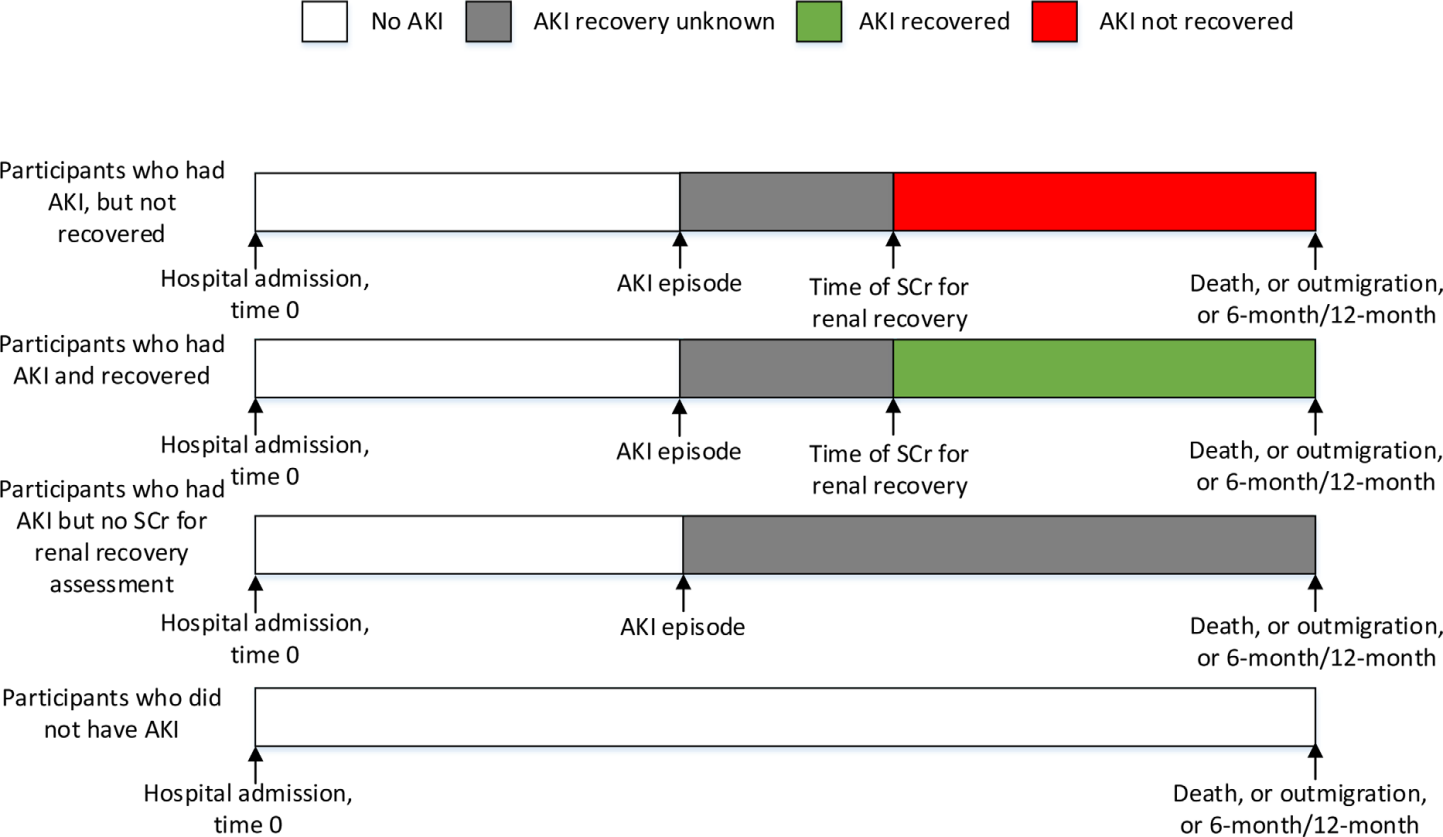
Timeline of exposure status updated by AKI episodes and renal recovery. Time of an AKI episode is the time of peak in-hospital SCr met AKI criteria during hospitalization. Time of SCr for AKI recovery is the time of closet SCr to 30 days post an AKI episode.

In the models fit for assessing the association between renal recovery and mortality, the composite of AKI episode and renal recovery were used to update exposure status during the course of follow-up. A person would contribute person time to the no AKI hazard before their AKI episode and person time to the AKI recovery unknown hazard after their AKI episode until the time of renal recovery assessment. After that time, they would contribute person time to the hazard of either AKI recovered or not recovered depending on whether the recovery SCr met the renal recovery requirement. If a person did not have a recovery SCr, then he would continue to contribute person time to the AKI recovery unknown hazard. Fig 1 illustrates how the exposure status were defined after each point of time. Patients were censored if they moved out of the province, or reached 6 months/12months from hospital admission.

Statistical analyses were performed using STATA MP 13 software (StataCorp, College Station, TX).

## Results

### Patient characteristics

**Fig 2:**
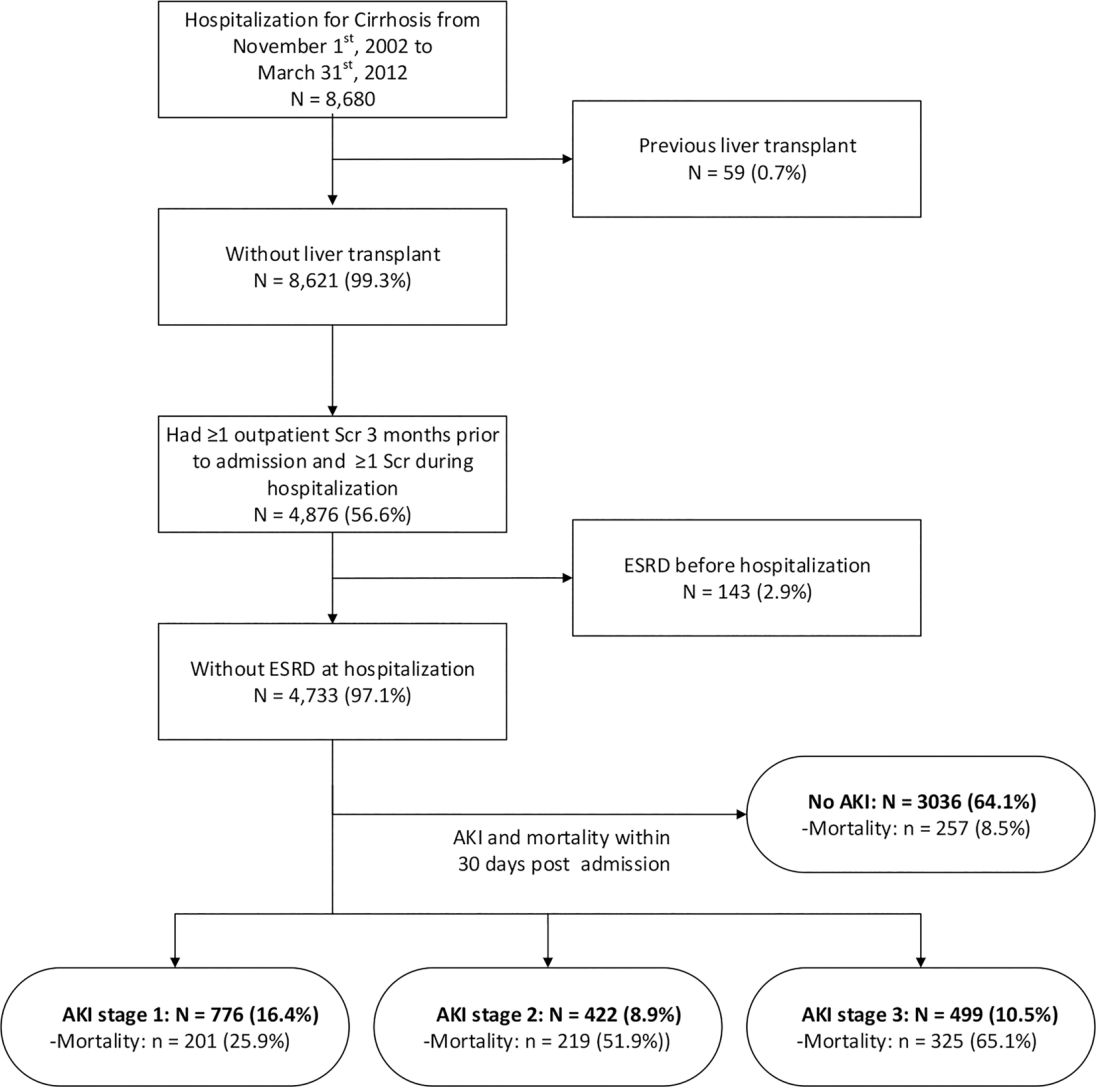
Study flow chart. SCr, serum creatinine; ESRD, end-stage renal disease; AKI, acute kidney injury.

Between November 1, 2002 and March 31, 2012, a total of 8,680 patients were hospitalized in the province of Alberta with a diagnosis of cirrhosis. Of these, 4,733 met all study inclusion criteria (Fig 2). The characteristics of the study cohort are outlined in Table 1. The median hospital length of stay was 8 (25^th^-75^th^ percentile, 4–19) days with a median duration of follow-up of 13.4 months (range 0.03–113.9 months; 25^th^ percentile, 1.4 months; 75^th^ percentile, 37.2 months). Overall, 1.0% of study participants were lost to follow-up due to out migration from the province. The mean time between hospital admission and the serum creatinine measurements used to determine recovery, or post discharge renal status in the case of the AKI participants are presented in S1 Table.

**Table 1:** Characteristics of the cohort according AKI stages.

Demographics	No AKI	AKI	P-value*	AKI stage1	AKI stage 2	AKI stage 3
Number of subjects (%)	2,883(60.9)	1,850(39.1)		812(17.2)	470(9.9)	568(12)
Age, mean (SD)	60.4(13.3)	61.5(12.9)	0.01	63.3(13.2)	61.6(12.5)	58.9(12.2)
Gender, male (%)	64.3	64.5	0.88	65.1	63.4	64.61
**Comorbid disease (%)**						
Myocardial infarction	7.3	13.2	<0.01	14.5	10.0	14.1
Peripheral vascular disease	8.8	10.1	0.12	10.3	8.7	10.9
Cerebrovascular disease	10.7	10.4	0.74	9.2	12.6	10.2
Congestive heart failure	18.9	29.0	<0.01	31.9	26.6	26.8
Diabetes Uncomplicated	23.0	18.0	<0.01	20.4	17.4	15.0
Diabetes Complicated	11.6	19.4	<0.01	18.8	19.1	20.4
Non-dermatologic malignancy	29.5	31.1	0.25	30.5	32.6	30.6
Chronic Pulmonary Disease	29.2	35.5	<0.01	36.1	37.0	33.3
Dementia	6.2	7.0	0.29	9.1	5.1	5.6
AIDS/HIV	1.6	1.8	0.56	1.8	1.7	1.8
Metastatic Carcinoma	8.5	10.9	0.01	9.4	11.3	12.9
Paraplegia and Hemiplegia	1.4	1.4	1.00	0.7	3.0	0.9
Peptic Ulcer Disease	16.5	19.5	0.01	20.3	20.2	17.6
Connective Tissue Disease-Rheumatic Disease	3.3	4.2	0.12	5.0	3.6	3.3
Mean CCI score (SD)	5.1(3)	6.1(3.3)	<0.01	6.1(3.3)	6.1(3.3)	6.3(3.3)
Baseline eGFR in mL/min/1.73^2^, mean(SD)	83.5(26.8)	76.3(29.4)	<0.01	71(71.2)	79.9(84.1)	81(85.6)

*p-value is for comparing AKI and no AKI

This table reflects AKI occurring during the entire hospitalization stay.

A total of 1850 (39%) of patients experienced an episode of AKI during their hospital stay. The majority of AKI episodes occurred during the first 30 days and 90 days of the hospital stay (1697(92%) and 1829(99%)). Participants with AKI had greater comorbidity (see Table 1) and a higher acuity of illness. Baseline kidney function was lower in participants who experienced AKI (mean eGFR 76.3 ml/min per 1.73 m^2^) than those who did not (mean eGFR 83.5 ml/min per 1.73 m^2^). Similar patient and kidney function parameters were seen when a sensitivity analysis was done using at least 2 diagnosis codes for cirrhosis (S2 Table).

### Impact of varying definitions of baseline renal function on AKI incidence

The observed incidence of AKI varied with the definition of baseline kidney function (Table 2). The incidence of AKI was lowest when the admission SCr was used and highest when the lowest SCr within the prior three months was used. The baseline SCr levels varied inversely with the incidence of AKI, with higher baseline SCr levels associated with lower AKI incidence and lower AKI severity (Table 2). When considering the impact of varying definitions of baseline renal function on the incidence of AKI within the first 30 days of hospitalization, a similar relationship was found (S3 and S4 Tables).

**Table 2:** Incidence of AKI by varying definitions of baseline renal function.

Baseline SCr	All subjects	no AKI	AKI stage1	AKI stage 2	AKI stage 3
**Number of subjects**					
3-month average	4,733	2883(60.9%)	812(17.2%)	470(9.9%)	568(12%)
Lowest SCr within 3 months	4,733	2659(56.2%)	891(18.8%)	525(11.1%)	658(13.9%)
6-month average	4,733	2850(60.2%)	824(17.4%)	479(10.1%)	580(12.3%)
Closet SCr to admission	4,733	2939(62.1%)	812(17.2%)	428(9%)	554(11.7%)
First SCr during hospitalization	4,733	3642(77%)	517(10.9%)	228(4.8%)	346(7.3%)
**Baseline SCr in μmol/L, mean(sd)**					
3-month average	90(44.6)	85.1(38.4)	105.7(56.6)	90.6(43.3)	92.2(50)
Lowest SCr within 3 months	83.5(38.9)	80(34.5)	93.6(47.1)	83.6(37.6)	83.9(42)
6-month average	88.8(42.2)	84(36.5)/0.95(0.41)	103.1(53.4)	91.4(42.6)	90.2(45.2)
Closet SCr to admission	93.1(54)	88.9(47.2)	110.6(74.2)	86.7(38.4)	94.3(57.7)
First SCr during hospitalization	112.8(88)	105.5(76)	141.1(114.3)	100.9(46.5)	155.7(143)
**Baseline SCr in mg/dL, mean(sd)**					
3-month average	1.02(0.5)	0.96(0.43)	1.2(0.64)	1.02(0.49)	1.04(0.57)
Lowest SCr within 3 months	0.94(0.44)	0.91(0.39)	1.06(0.53)	0.95(0.42)	0.95(0.47)
6-month average	1(0.48)	0.95(0.41)	1.17(0.6)	1.03(0.48)	1.02(0.51)
Closet SCr to admission	1.05(0.61)	1.01(0.53)	1.25(0.84)	0.98(0.43)	1.07(0.65)
First SCr during hospitalization	1.28(1)	1.19(0.86)	1.6(1.29)	1.14(0.53)	1.76(1.62)

For an exact conversion from μmol/L of creatinine to mg/dL of creatinine, multiply by 0.0113

### Association between AKI and mortality

**Fig 3:**
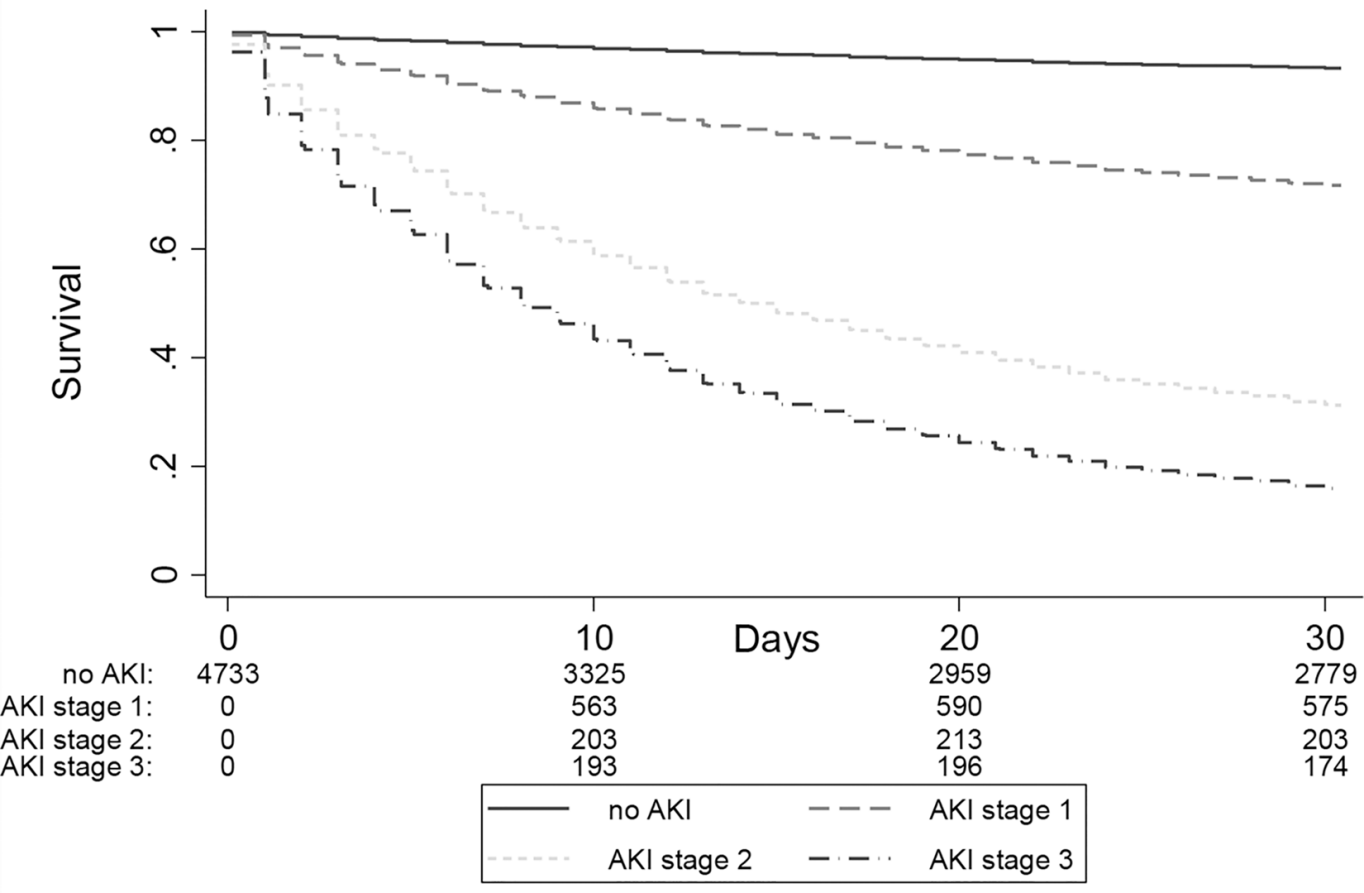
30-day mortality according to AKI stages. The 30-day mortality increased with increasing AKI severity. The numbers at the bottom of the survival curves represent the number of patients at risk at various time points.

AKI was strongly associated with mortality. The overall 30-day mortality was higher for participants with AKI compared to those without AKI (43.9% vs 8.5%; p-value<0.001) (Fig 3) and increased with increasing AKI severity (Table 3). As compared to a reference group of participants who did not develop AKI, the hazard ratio for 30-day mortality increased from 4.8 to 16.8 to 26.5 in AKI-stages 1, 2 and 3, respectively. In the sensitivity analysis of 1115 patients with at least 2 diagnostic codes for cirrhosis, the hazard ratios for 30-day mortality increased from 5.8 to 41.5 in AKI stages 1–3, when compared to those participants who did not develop AKI (S5 Table), which suggests that the mortality risk associated with AKI may be even stronger in patients meeting more stringent diagnostic coding requirements for cirrhosis. As compared to a reference group of participants who did not develop AKI, AKI stage 1 with a maximum SCr ≤ 132 μmol/L was associated with a significant 3.5 fold increased hazard of death at 30 days (Table 4).

**Table 3:** Hazard ratios (95% CI) for mortality according to AKI stages (N = 4,733).

	30 day mortality	90 day mortality
*No of event (%)*	1002(21.2%)	1569(33.2%)
No AKI	1[reference]	1[reference]
AKI stage 1	4.81(3.98, 5.8)	3.22(2.8, 3.71)
AKI stage 2	16.75(13.94, 20.13)	9.37(8.09, 10.85)
AKI stage 3	26.48(22.34, 31.38)	15.74(13.78, 17.97)

Model was adjusted for age, gender, comorbid disease (MI, PVD, CEVD, CHF, diabetes uncomplicated/complicated, cancer, COPD, dementia, AIDS/HIV, metastatic carcinoma, paraplegia and hemiplegia, peptic ulcer disease, connective tissue disease, and rheumatic disease), paracentesis and esophageal varices with bleeding during hospitalization, and baseline kidney function(baseline eGFR).Follow-up started from hospital admission date for all participants. AKI episodes were treated as a time-varying exposure.

**Table 4:** Hazard ratios for mortality according to AKI with or without Scr >132 mmol/l (N = 4,733).

	30 day mortality	90 day mortality
*No of event (%)*	1002(21.2%)	1569(33.2%)
No AKI	1[reference]	1[reference]
AKI stage 1	3.52(2.63, 4.72)	2.54(2.04, 3.18)
AKI stage 2	5.66(4.56, 7.03)	3.64(3.09, 4.3)
AKI stage 3	21.28(18.26, 24.78)	12.29(10.94, 13.81)

Model was adjusted for age, gender, comorbid disease(MI, PVD, CEVD, CHF, diabetes uncomplicated/complicated, cancer, COPD, DEMENTIA, AIDS/HIV, metastatic carcinoma, paraplegia and hemiplegia, peptic ulcer disease, connective tissue disease, and rheumatic disease), paracentesis and esophageal varices with bleeding during hospitalization, and baseline kidney function(baseline eGFR).Follow-up started from hospital admission date for all participants. AKI episodes were treated as a time-varying exposure.

A sensitivity analysis was performed to determine the impact of different definitions of baseline renal function on mortality and is presented as a forest plot in S6 Table. The hazard ratios for mortality were highest when using the SCr on the day of admission (hazard ratio of 7.2 for AKI stage 1). Importantly, the hazard ratios associated with using the other three definitions of baseline creatinine were similar (4.2–4.8).

### Recovery of renal function

**Fig 4:**
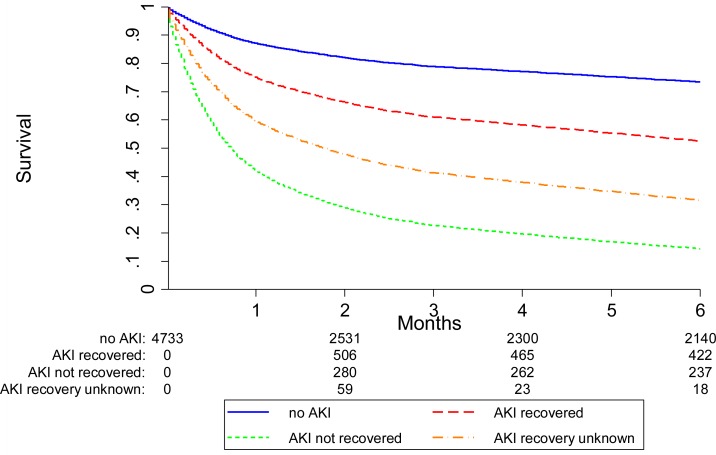
6-month mortality according to AKI recovery (as a time-varying covariate with follow-up from hospital admission for all subjects). Mortality was lowest in those patients without AKI, higher in who recovered renal function and highest in those patients without renal recovery. The numbers at the bottom of the survival curves represent the number of patients at risk in each AKI recovery group at various time points.

**Fig 5:**
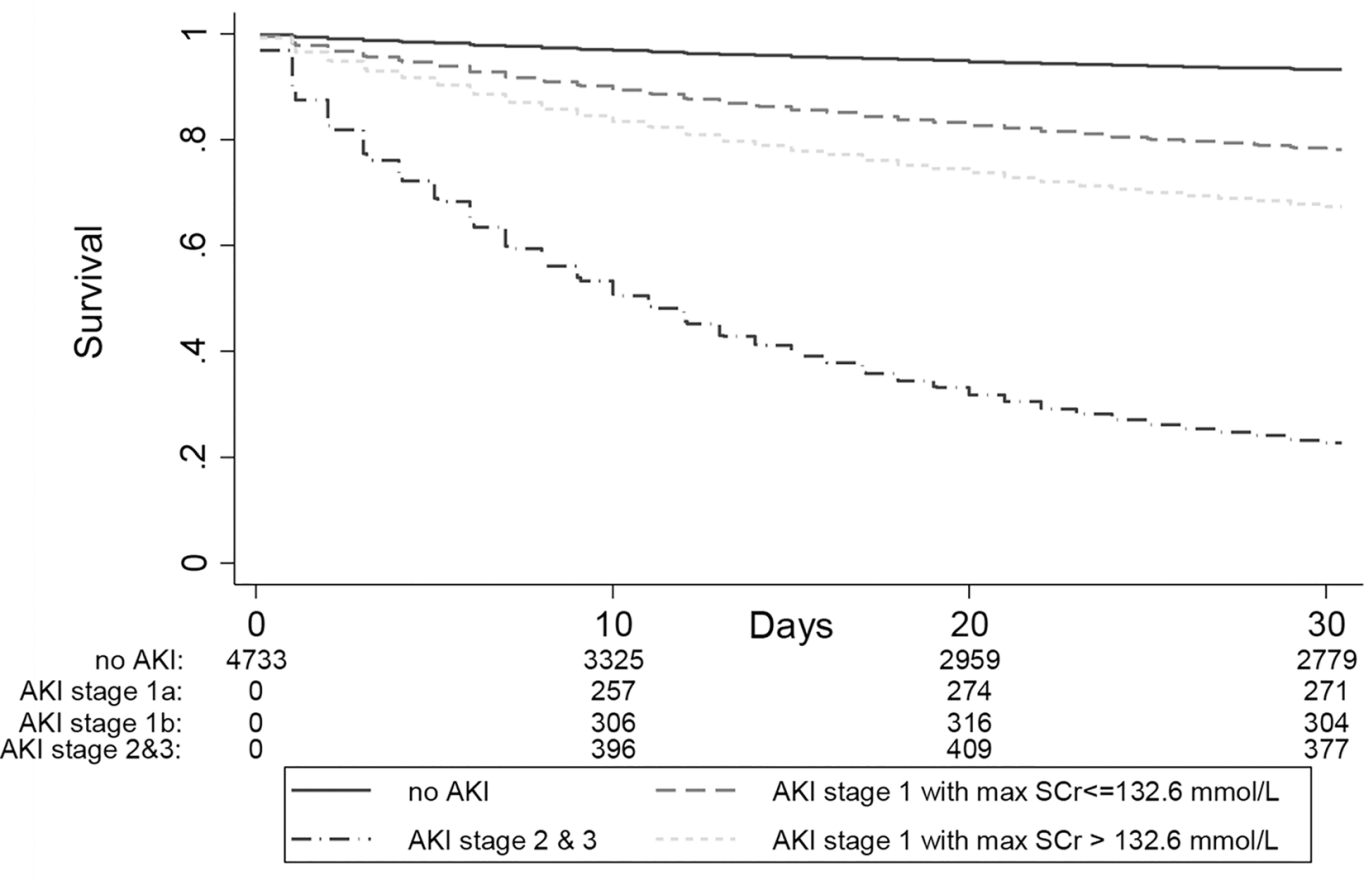
30-day mortality according to AKI with or without Scr >132.6 mmol/l. The numbers at the bottom of the survival curves represent the number of patients at risk in each AKI and renal recovery group at various time points. (AKI stage 1a: AKI stage 1 with max SCr< = 132.6 μmol/L; AKI stage 1b: AKI stage 1 with max SCr>132.6 μmol/L)

The overall mortality of the cohort was 40.2% at 6 months. Of the 1375 patients with AKI during the first 6 months after hospital admission in whom recovery could be assessed, 667 (48.5%) recovered to within 25% of their baseline SCr. Hazard ratios for 6 and 12 month mortality according to renal recovery status are presented in Table 5. In AKI survivors who recovered renal function, 63% were alive at 6-months compared with 33% of AKI survivors who did not recover (Figs 4 and 5).

**Table 5:** Hazard ratio for mortality according to renal recovery status (N = 4,733, AKI and renal recovery as time varying covariate).

	6-month mortality	12-month mortality
*No of event (%)*	1905(40.2%)	2235(47.2%)
**No AKI**	1[reference]	1[reference]
**AKI recovered**	2.1(1.8, 2.45)	1.79(1.57, 2.06)
**AKI not recovered**	6.34(5.54, 7.26)	4.61(4.07, 5.24)
**AKI recovery unknown**	3.86(3.42, 4.35)	3.64(3.23, 4.09)

Model was adjusted for age, gender, comorbid disease(MI, PVD, CEVD, CHF, diabetes uncomplicated/complicated, cancer, COPD, DEMENTIA, AIDS/HIV, metastatic carcinoma, paraplegia and hemiplegia, peptic ulcer disease, connective tissue disease, and rheumatic disease), paracentesis and esophageal varices with bleeding during hospitalization, and baseline kidney function(baseline eGFR).Follow-up started from hospital admission date for all participants. AKI episodes and renal recovery were treated as a time-varying exposure.

## Discussion

Several important findings were identified by our analysis of this large retrospective cohort of hospitalized patients with cirrhosis. First, in keeping with recent studies, AKI was a common complication during hospitalization, occurring in 39% of patients with cirrhosis. Second, the way that baseline kidney function was defined had significant implications on the incidence of AKI in cirrhotic patients; fluctuations in kidney function are frequent in these patients and use of admission SCr values likely underestimates the true incidence of AKI. Third, in keeping with what has been found in non-cirrhotic populations, a diagnosis of AKI stage 1 increased the hazard of death at 30 days. Finally, AKI had prognostic significance despite renal recovery. Those patients who recovered their renal function to within 25% of the baseline SCr within 30 days had a significant increase in both 6 month and, on sensitivity analysis, 12 month mortality.

A baseline SCr is required for the diagnosis of AKI by recent consensus [20] criteria [20]. Previous studies of patients with cirrhosis have been inconsistent in their approach, ranging from using i) the most recent stable reading prior to admission [14], ii) an average of SCr measurements between 7–90 days before admission [9], iii) a baseline SCr within 3 months before admission [2] and iv) day of admission SCr values [8]. Current guidelines are based on expert consensus. The Acute Dialysis Quality initiative-International Ascites Club working group had originally suggested that a stable SCr reading taken within 6 months be used to define baseline [2, 15]. More recently, the need for “application of a more rigorous time frame for the definition of AKI” have resulted in a revision to “the use of the last value of the SCr within the last 3 months before admission” [7].

The current study raises two main points with regards to choice of baseline SCr. First, our data demonstrated a 47% reduction in the reported incidence of AKI with the use of day of admission creatinine values. This is in keeping with non-cirrhosis cohorts [6, 10, 24] where surrogates for pre-admission creatinine values lead to a bidirectional misclassification of AKI incidence and prognosis. Therefore, pre-admission SCr values should be used preferentially over day of admission values. When we evaluated the impact of varying definitions of baseline renal function on the hazard ratio for mortality, the hazard for the closest pre-admission SCr and the lowest SCr within 3 months prior to admission were similar. Using a large external cohort of patients, for the first time, the current data therefore serves to validate the choice of baseline SCr (closest pre-admission SCr within 3 months) made by the recent expert-based consensus guidelines [7]. The use of this definition in future studies will help to maintain consistency in clinical practice and research.

As has been observed in other cohorts there was a graded association between peak AKI stage and mortality, the magnitude of risk at each AKI stage consistent with and in fact higher than that observed in multiple non-cirrhosis cohorts [25–33]. This may be due in part to the intertwined pathophysiology of AKI and liver disease–as observed in hepatorenal syndrome, where AKI may also be a marker of worsening liver function. Although not assessable with our administrative dataset, the discordance in mortality rates is likely explained by variability in the threshold for hospitalization and the variation in liver disease severity admitted to each institution. Moreover, the reliance on administrative codes for cirrhosis identification can bias selection towards a sicker patient population. Lastly, unlike studies using in-hospital mortality as the outcome [8, 14], by using 30-day mortality (and 90-day mortality on sensitivity analysis), our study had a longer follow-up time in which to ascertain deaths.

The findings of this study supports the relevance of AKI stage 1 independent of whether a threshold creatinine of 132.6 μmol/L [8, 9] was reached. Patients with AKI stage 1 and a SCr of ≤ 132.6 μmol/L had a 3.5 times increased hazard for death compared to those patients without AKI. SCr is an unreliable estimate of stable kidney function in cirrhosis and single readings may be even less reliable in the setting of AKI. Due to reduced creatine synthesis by the liver, reduced creatinine generation due to muscle wasting, and an increased volume of distribution SCr may in fact overestimate kidney function by as much as 100% [6]. The use of SCr based equations for estimating glomerular filtration rate (GFR) have not been validated in populations with cirrhosis. In the absence of a reliable marker of kidney function and/or changes in kidney function, using a combination of absolute and relative changes in SCr as recommended in recent international guidelines defining AKI allows earlier detection of significant kidney injury in cirrhosis and avoids the risk of delaying any potential therapeutic interventions. This is particularly relevant in the setting of cirrhosis since the response to vasoconstrictor therapy decreases with higher SCr levels at treatment initiation.

Consistent with other non-cirrhotic and cirrhotic cohorts, the patients in the current series who did not recover renal function within 30 days had a 5–6 fold increase in mortality as compared to patients who did not develop AKI. Importantly, even complete renal recovery (SCr to within 25% of the baseline value within 30 days) was a marker for a worse prognosis, associated with a two-fold increase in the risk of either 6-month or 12-month mortality. The prognostic relevance of complete renal recovery in the non-cirrhosis literature has been discordant [34, 35]. The data on renal recovery in patients with cirrhosis has been limited. Wong *et al*. reported a 15% 30-day mortality rate in patients with complete renal recovery (defined as SCr returning to pre-AKI level at the end of the AKI episode). At present, our data supports that transient changes in renal function are a marker for poor prognosis and should not be dismissed. Intensive management should be directed to preventing AKI in patients with cirrhosis.

Several study limitations warrant mention. Although all observational studies are vulnerable to residual confounding, our study adjusted for several important confounders, including a comorbidity index. A second limitation is ascertainment bias inherent in the use of laboratory data to identify AKI. In keeping with previous studies, patients who had SCr values available before the admission period represent a sicker population of patients (S7 Table). The exclusion of the patients without prior creatinine values who likely have normal or near-normal kidney function may overestimate the reported effect estimates in this group. As with all recovery analyses, a noted limitation is that the impact of renal recovery could only be assessed in the subset of patients with follow-up renal data within the 30-day recovery assessment period. Moreover, we did not have access to liver function or liver disease etiology variables in our database and therefore were unable to adjust for these in our analyses. Finally, the use of administrative data does not allow assessment of AKI etiology, which may influence the likelihood of renal recovery and long-term outcomes. Previous studies of AKI in hospitalized patients with cirrhosis [1] would suggest that prerenal causes account for the vast majority of cases, followed by ATN. The findings of this study may apply best to these diagnoses.

In conclusion, in a large population based cohort of hospitalized patients with cirrhosis, we confirmed the graded increases in mortality associated with increasing severity of AKI. This study extends the results of previous evaluations by highlighting the impact that different approaches to defining baseline SCr have on recognition of AKI. Moreover, the results support the prognostic relevance of a diagnosis of AKI stage 1 even when peak SCr remains below 132.6 μmol/L and increased risk of long-term mortality even when there is a recovery of renal function within 30 days of the AKI episode.

## Supporting Information

S1 Table Days of SCr drawn used to determine renal status within 30 days after AKI event or 30 days post discharge in the case of non-AKI. (DOCX)

S2 Table Patients’ characteristic according AKI stages (for subjects with ≥2 diagnosis code for cirrhosis). (DOCX)

S3 Table Incidence of AKI within 30 days of hospital admission by varying definitions of baseline renal function. (DOCX)

S4 Table Comparison of AKI stages within 30 days of hospital admission defined by closet SCr to admission and lowest SCr within 3 months of admission, number of patients (%). (DOCX)

S5 Table Hazard ratios (95% CI) for mortality according to AKI stages (N = 1,115, for subjects with ≥2 diagnosis code for cirrhosis). Model was adjusted for age, gender, comorbid disease (MI, PVD, CEVD, CHF, diabetes uncomplicated/complicated, cancer, COPD, DEMENTIA, AIDS/HIV, metastatic carcinoma, paraplegia and hemiplegia, peptic ulcer disease, connective tissue disease, and rheumatic disease), paracentesis and esophageal varices with bleeding during hospitalization, and baseline kidney function(baseline eGFR). Follow-up started from hospital admission date for all participants. AKI episodes were treated as a time-varying exposure.
(DOCX)

S6 Table Forest plot demonstrating the hazard ratios for mortality according to AKI stages by varying definitions of baseline renal function. (DOCX)

S7 Table Comparison between the patients without SCr in the 3 months before and during hospitalization to final cohort. (DOCX)
