# Supplementary material for: Relevance of New Definitions to Incidence and Prognosis of Acute Kidney Injury in Hospitalized Patients with Cirrhosis: A Retrospective Population-Based Cohort Study
Source: PLoS One. 2016 Aug 9;11(8):e0160394. doi: 10.1371/journal.pone.0160394 (PMC4978466; doi:10.1371/journal.pone.0160394)
Supplement: S1 Table — (DOCX) [file pone.0160394.s001.docx]

**S1 Table**

|  | **Mean** | **SD** | **No of subject with ≥1 SCr*** |
| --- | --- | --- | --- |
| no AKI | 19.8 | 8.4 | 1612 |
| AKI | 17.1 | 11.1 | 1382 |

* Number of patients who had ≥1 Serum Creatinine measurements within 30 days after AKI episodes or within 30 days post discharge in the case of non-AKI. The means and standard deviations were calculated based on the 1612 and d1382 patients.
