# Supplementary material for: Relevance of New Definitions to Incidence and Prognosis of Acute Kidney Injury in Hospitalized Patients with Cirrhosis: A Retrospective Population-Based Cohort Study
Source: PLoS One. 2016 Aug 9;11(8):e0160394. doi: 10.1371/journal.pone.0160394 (PMC4978466; doi:10.1371/journal.pone.0160394)
Supplement: S2 Table — (DOCX) [file pone.0160394.s002.docx]

**S2 Table**

| **Demographics** | **No AKI** | **AKI** | **P-value*** | **AKI stage1** | **AKI stage 2** | **AKI stage 3** |
| --- | --- | --- | --- | --- | --- | --- |
| Number of subjects (%) | 580(52) | 535(48) |  | 189(17) | 136(12.2) | 210(18.8) |
| Age, mean (SD) | 58.1(13.8) | 59.9(12.9) | 0.02 | 63.1(13.3) | 60.3(12.8) | 56.7(11.8) |
| Male gender(n[%]) | 62.6 | 63.9 | 0.64 | 66.7 | 66.2 | 60.00 |
|  |  |  |  |  |  |  |
| **Comorbid disease (%)** |  |  |  |  |  |  |
| Myocardial infarction | 5.0 | 11.8 | <0.01 | 13.8 | 10.3 | 10.95 |
| Peripheral vascular disease | 7.9 | 7.9 | 0.96 | 9.0 | 5.9 | 8.10 |
| Cerebrovascular disease | 8.6 | 8.0 | 0.73 | 4.8 | 9.6 | 10.00 |
| Congestive heart failure | 14.5 | 24.5 | <0.01 | 31.7 | 20.6 | 20.48 |
| Diabetes Uncomplicated | 25.9 | 18.3 | <0.01 | 20.1 | 21.3 | 14.76 |
| Diabetes Complicated | 10.7 | 19.3 | <0.01 | 18.0 | 19.9 | 20.00 |
| Non-dermatologic malignancy | 25.7 | 24.7 | 0.70 | 24.3 | 28.7 | 22.38 |
| Chronic Pulmonary Disease | 23.6 | 34.0 | <0.01 | 33.3 | 33.1 | 35.24 |
| Dementia | 4.3 | 7.5 | 0.02 | 11.1 | 2.9 | 7.14 |
| AIDS/HIV | 1.0 | 2.1 | 0.16 | 2.1 | 0.7 | 2.86 |
| Paraplegia and Hemiplegia | 1.4 | 0.9 | 0.49 | 0.0 | 2.2 | 0.95 |
| Peptic Ulcer Disease | 19.7 | 25.0 | 0.03 | 27.0 | 30.9 | 19.52 |
| Connective Tissue Disease | 3.4 | 4.9 | 0.24 | 5.8 | 3.7 | 4.76 |
| Mean CCI score (SD) | 5.6(2.8) | 6.3(2.9) | <0.01 | 6.4(2.8) | 6.3(3.1) | 6.1(2.8) |
| Baseline eGFR in mL/min/1.73^2^, mean(SD) | 86.3(26.6) | 77.6(29.2) | 0.035 | 70.1(69.3) | 76.9(79.3) | 84.7(89.4) |

* p-value is for comparing AKI and no AKI
