# Supplementary material for: Relevance of New Definitions to Incidence and Prognosis of Acute Kidney Injury in Hospitalized Patients with Cirrhosis: A Retrospective Population-Based Cohort Study
Source: PLoS One. 2016 Aug 9;11(8):e0160394. doi: 10.1371/journal.pone.0160394 (PMC4978466; doi:10.1371/journal.pone.0160394)
Supplement: S3 Table — (DOCX) [file pone.0160394.s003.docx]

**S3 Table**

| **Baseline SCr** | **All subjects** | **no AKI** | **AKI stage1** | **AKI stage 2** | **AKI stage 3** |
| --- | --- | --- | --- | --- | --- |
| **Number of subjects** | |  |  |  |  |
| 3-month average | 4,733 | 3036(64.2%) | 776(16.4%) | 422(8.9%) | 499(10.5%) |
| Lowest SCr within 3 months | 4,733 | 2822(59.6%) | 854(18%) | 473(10%) | 584(12.3%) |
| 6-month average | 4,733 | 3004(63.5%) | 789(16.7%) | 430(9.1%) | 510(10.8%) |
| Closet SCr to admission | 4,733 | 3096(65.4%) | 772(16.3%) | 379(8%) | 486(10.3%) |
| First SCr during hospitalization | 4,733 | 3790(80.1%) | 473(10%) | 182(3.9%) | 288(6.1%) |
| **Baseline SCr in µmol/L, mean(sd)** | |  |  |  |  |
| 3-month average | 90(44.6) | 85.1(38.4) | 105.7(56.6) | 90.6(43.3) | 92.2(50) |
| Lowest SCr within 3 months | 83.5(38.9) | 80.3(34.9) | 93.3(47) | 83.8(37.7) | 84.5(42.7) |
| 6-month average | 93.6(51.3) | 85.3(38.7) | 105.9(56.9) | 93.6(51.3) | 88.8(42.2) |
| Closet SCr to admission | 93.1(54) | 84.2(36.7) | 103.2(53.6) | 91.3(46.3) | 93.1(54) |
| First SCr during hospitalization | 112.8(88) | 105.2(75.2) | 144.3(117.8) | 102.1(46.8) | 167.8(152.3) |
| **Baseline SCr in mg/dL*, mean(sd)** | |  |  |  |  |
| 3-month average | 0.97(0.44) | 0.95(0.42) | 1.17(0.61) | 1.04(0.48) | 1.03(0.52) |
| Lowest SCr within 3 months | 0.94(0.44) | 0.91(0.39) | 1.06(0.53) | 0.95(0.43) | 0.96(0.48) |
| 6-month average | 1(0.48) | 0.95(0.42) | 1.17(0.61) | 1.04(0.48) | 1.03(0.52) |
| Closet SCr to admission | 1.05(0.61) | 1.01(0.53) | 1.26(0.85) | 0.98(0.43) | 1.09(0.68) |
| First SCr during hospitalization | 1.28(1) | 1.19(0.85) | 1.63(1.33) | 1.15(0.53) | 1.9(1.72) |
| **Number of days from closet SCr to admission date, mean(sd)** | | | | | |
|  | 25.5(24.3) | 24.8(24.3) | 25.2(24) | 27.6(24) | 28.3(25.3) |

* For an exact conversion from µmol/L of creatinine to mg/dL of creatinine, multiply by 0.0113.
