# Supplementary material for: Relevance of New Definitions to Incidence and Prognosis of Acute Kidney Injury in Hospitalized Patients with Cirrhosis: A Retrospective Population-Based Cohort Study
Source: PLoS One. 2016 Aug 9;11(8):e0160394. doi: 10.1371/journal.pone.0160394 (PMC4978466; doi:10.1371/journal.pone.0160394)
Supplement: S4 Table — (DOCX) [file pone.0160394.s004.docx]

**S4 Table**

|  |  | **AKI stages by closest SCr to admission*** | | | | |
| --- | --- | --- | --- | --- | --- | --- |
|  |  | **0** | **1** | **2** | **3** | **Total** |
| **AKI stages by lowest SCr within 3 months** | **0** | 2822(59.6%) | 0(0%) | 0(0%) | 0(0%) | 2822(59.6%) |
|  | **1** | 220(4.6%) | 634(13.4%) | 0(0%) | 0(0%) | 854(18%) |
|  | **2** | 45(1%) | 98(2.1%) | 330(7%) | 0(0%) | 473(10%) |
|  | **3** | 9(0.2%) | 40(0.8%) | 49(1%) | 486(10.3%) | 584(12.3%) |
|  | **Total** | 3096(65.4%) | 772(16.3%) | 379(8%) | 486(10.3%) | 4,733 |

* Kappa = 0.826, p-value<0.001
