# Supplementary material for: Relevance of New Definitions to Incidence and Prognosis of Acute Kidney Injury in Hospitalized Patients with Cirrhosis: A Retrospective Population-Based Cohort Study
Source: PLoS One. 2016 Aug 9;11(8):e0160394. doi: 10.1371/journal.pone.0160394 (PMC4978466; doi:10.1371/journal.pone.0160394)
Supplement: S5 Table — Model was adjusted for age, gender, comorbid disease (MI, PVD, CEVD, CHF, diabetes uncomplicated/complicated, cancer, COPD, DEMENTIA, AIDS/HIV, metastatic carcinoma, paraplegia and hemiplegia, peptic ulcer disease, connective tissue disease, and rheumatic disease), paracentesis and esophageal varices with bleeding during hospitalization, and baseline kidney function(baseline eGFR). Follow-up started from hospital admission date for all participants. AKI episodes were treated as a time-varying exposure. (DOCX) [file pone.0160394.s005.docx]

**S5 Table**

|  | **30 day mortality** | **90 day mortality** |
| --- | --- | --- |
| ***No of event (%)*** | 286(25.7%) | 434(38.9%) |
| No AKI | 1[reference] | 1[reference] |
| AKI stage 1 | 5.83(3.89, 8.75) | 3.52(2.6, 4.76) |
| AKI stage 2 | 20.52(13.92, 30.25) | 11.47(8.52, 15.44) |
| AKI stage 3 | 41.46(29.09, 59.09) | 23.93(18.37, 31.18) |
