# Supplementary material for: Relevance of New Definitions to Incidence and Prognosis of Acute Kidney Injury in Hospitalized Patients with Cirrhosis: A Retrospective Population-Based Cohort Study
Source: PLoS One. 2016 Aug 9;11(8):e0160394. doi: 10.1371/journal.pone.0160394 (PMC4978466; doi:10.1371/journal.pone.0160394)
Supplement: S7 Table — (DOCX) [file pone.0160394.s007.docx]

**S7 Table**

| **Demographics** | **Patients in final cohort** | **Patents without SCr in the 3 months before or during hospitalization^1^** | **p-value** |
| --- | --- | --- | --- |
| Number of subjects (%) | 4,733 | 3,733 |  |
| Age, mean (SD) | 60.8(13.1) | 58(14.7) | <0.01 |
| Gender, male (%) | 64.4 | 65.0 | 0.53 |
|  |  |  |  |
| **Comorbid disease (%)** |  |  |  |
| Myocardial infarction | 9.6 | 8.8 | 0.18 |
| Peripheral vascular disease | 9.3 | 6.6 | <0.01 |
| Cerebrovascular disease | 10.6 | 9.3 | 0.05 |
| Congestive heart failure | 22.8 | 16.6 | <0.01 |
| Diabetes Uncomplicated | 21.0 | 16.4 | <0.01 |
| Complicated | 14.6 | 8.6 | <0.01 |
| Non-dermatologic malignancy | 30.1 | 18.2 | <0.01 |
| Chronic Pulmonary Disease | 31.7 | 30.0 | 0.09 |
| Dementia | 6.5 | 5.9 | 0.20 |
| AIDS/HIV | 1.6 | 0.9 | 0.00 |
| Paraplegia and Hemiplegia | 1.4 | 1.6 | 0.33 |
| Peptic Ulcer Disease | 17.7 | 17.1 | 0.48 |
| Connective Tissue Disease-Rheumatic Disease | 3.6 | 2.5 | <0.01 |
| Mean CCI score (median, interquartile range) | 5.5(3.1) | 4.5(3) | <0.01 |
|  |  |  |  |
| **Procedure or condition during hospitalization (%)** | |  |  |
| Paracentesis | 23.9 | 19.0 | <0.01 |
| Oesophageal varices with bleeding | 4.4 | 6.4 | <0.01 |

**^1^** Population Included patients with hospitalization for cirrhosis form November 1^st^. 2002 to March 31^st^, 2012, and without liver transplant before hospitalization. 12 patients with ESRD (chronic dialysis or renal transplant) prior to hospitalization were excluded.
